# (±)-(4a*R*,5*R*,8*S*,8a*R*)-8-(*tert*-Butyl­dimethyl­sil­yloxy)-2,5,8a-trimethyl-4a,5,8,8a-tetra­hydro­naphthalene-1,4-dione

**DOI:** 10.1107/S1600536813002973

**Published:** 2013-02-02

**Authors:** Felix N. Delling, Julio Zukerman-Schpector, Timothy J. Brocksom, Ursula Brocksom, Fernanda G. Finelli, Edward R. T. Tiekink

**Affiliations:** a Department of Chemistry, Universidade Federal de São Carlos, 13565-905 São Carlos, SP, Brazil; b Department of Chemistry, University of Malaya, 50603 Kuala Lumpur, Malaysia

## Abstract

In the title compound, C_19_H_30_O_3_Si, both rings adopt a half-boat conformation. Overall, the mol­ecule approximates a U-shape as the *cyclo*-2-ene-1,4-dione and butyl­dimethyl­sil­yloxy substituents lie to the same side of the central cyclo­hexene ring; the methyl substituent lies to the other side of the mol­ecule. In the crystal, linear supra­molecular chains along the *b* axis are sustained by C—H⋯O inter­actions.

## Related literature

For a general description of the synthesis of higher terpenoids using the Diels–Alder reaction, see: Brocksom *et al.* (2001 ▶). For the synthesis of a similar compound containing an N atom in place of the O atom, see: Vieira *et al.* (2007 ▶). For the synthesis, see: Finelli (2004 ▶). For additional conformational analysis, see: Cremer & Pople (1975 ▶).
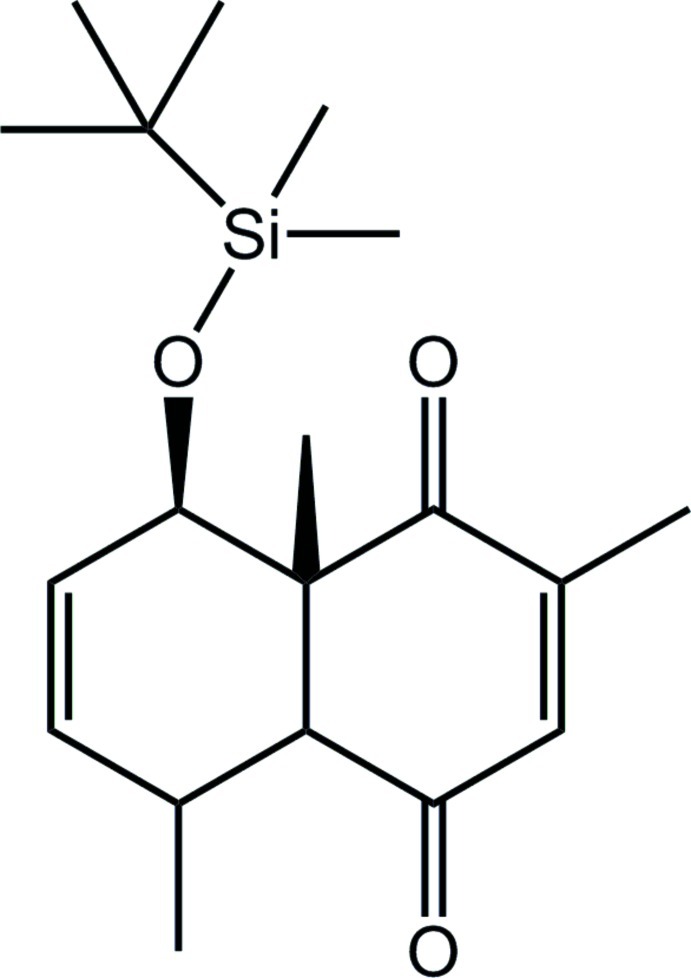


## Experimental

### 

#### Crystal data

C_19_H_30_O_3_Si
*M* *_r_* = 334.52
Monoclinic, 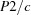

*a* = 15.325 (2) Å
*b* = 7.1744 (9) Å
*c* = 17.965 (2) Å
β = 93.577 (9)°
*V* = 1971.4 (4) Å^3^
*Z* = 4
Mo *K*α radiation
μ = 0.13 mm^−1^
*T* = 293 K
0.15 × 0.10 × 0.08 mm

#### Data collection

Enraf–Nonius CAD-4 MACH 3 diffractometer
4451 measured reflections
4305 independent reflections
1463 reflections with *I* > 2σ(*I*)
*R*_int_ = 0.072
3 standard reflections every 30 min intensity decay: 1.4%

#### Refinement

*R*[*F* ^2^ > 2σ(*F* ^2^)] = 0.062
*wR*(*F* ^2^) = 0.165
*S* = 0.93
4305 reflections
216 parameters
H-atom parameters constrained
Δρ_max_ = 0.17 e Å^−3^
Δρ_min_ = −0.21 e Å^−3^

### 

Data collection: *CAD-4 Software* (Enraf–Nonius, 1989 ▶); cell refinement: *CAD-4 Software*; data reduction: *MolEN* (Fair, 1990 ▶); program(s) used to solve structure: *SIR92* (Altomare *et al.*, 1999 ▶); program(s) used to refine structure: *SHELXL97* (Sheldrick, 2008 ▶); molecular graphics: *ORTEP-3 for Windows* (Farrugia, 2012 ▶), *DIAMOND* (Brandenburg, 2006 ▶) and *MarvinSketch* (ChemAxon, 2009 ▶); software used to prepare material for publication: *publCIF* (Westrip, 2010 ▶).

## Supplementary Material

Crystal structure: contains datablock(s) global, I. DOI: 10.1107/S1600536813002973/hg5287sup1.cif

Structure factors: contains datablock(s) I. DOI: 10.1107/S1600536813002973/hg5287Isup2.hkl

Supplementary material file. DOI: 10.1107/S1600536813002973/hg5287Isup3.cml

Additional supplementary materials:  crystallographic information; 3D view; checkCIF report

## Figures and Tables

**Table 1:** Hydrogen-bond geometry (Å, °)

*D*—H⋯*A*	*D*—H	H⋯*A*	*D*⋯*A*	*D*—H⋯*A*
C9—H9⋯O2^i^	0.98	2.55	3.524 (5)	171

Symmetry code: (i) 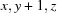.

**Fig. 1.**
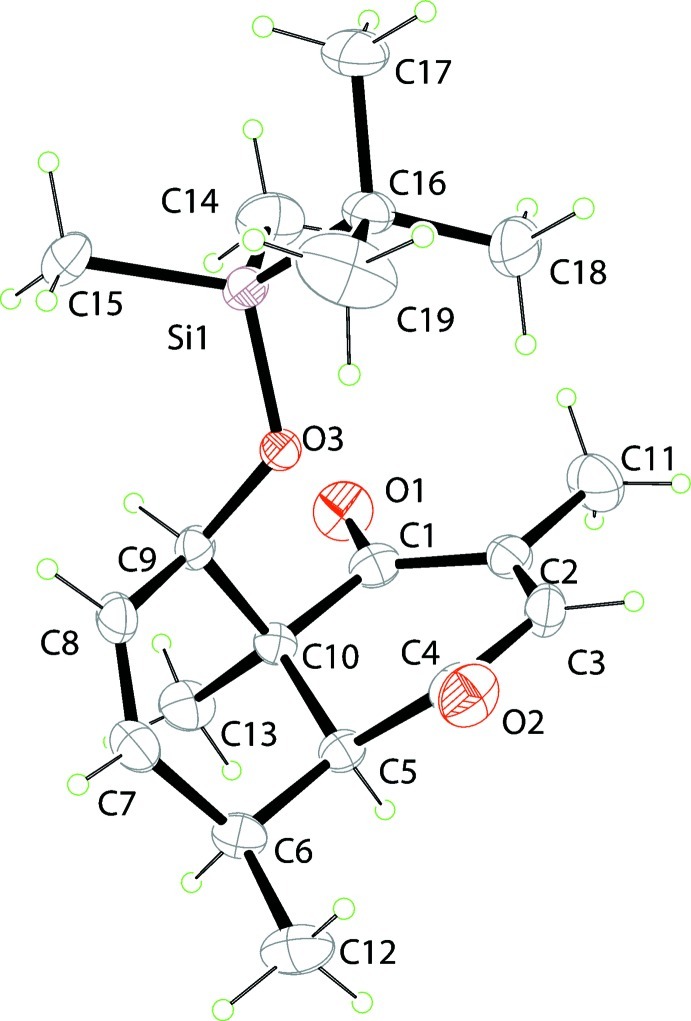
The molecular structure of compound (I) showing atom labelling scheme and displacement ellipsoids at the 50% probability level (arbitrary spheres for the H atoms).

**Fig. 2.**
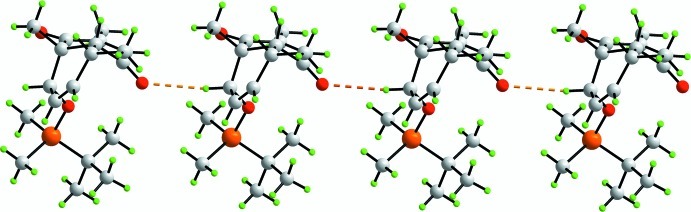
A view of the linear supramolecular chain sustained by C—H···O interactions (orange dashed lines) in the crystal structure of (I).

**Fig. 3.**
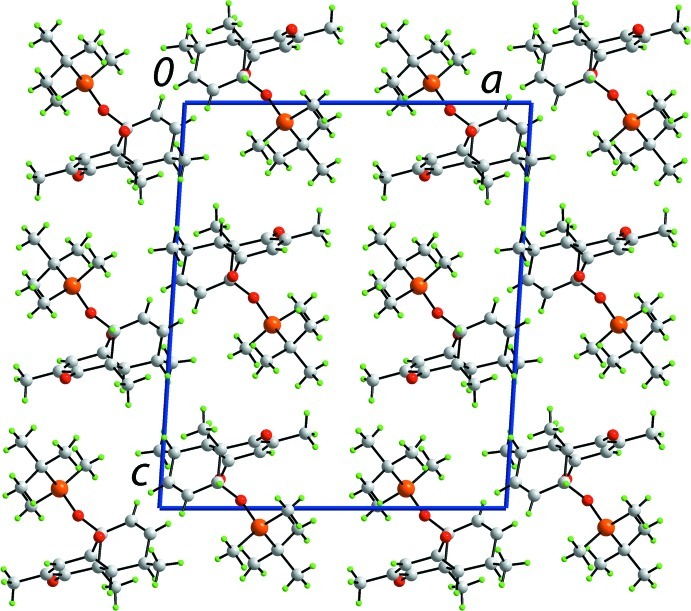
A view in projection down the b axis of the unit-cell contents of (I).

## supplementary crystallographic information

### Comment

The synthesis of polycyclic natural products such as sesqui- and di-terpenes frequently requires the construction of the decalin nucleus. This can be performed using the Diels-Alder reaction between appropriate acyclic dienes and monocyclic dienophiles such as *para*-benzoquinones. The desired appendages around the decalin nucleus can include oxygen and alkyl substituents, which are conveniently included in the diene and dienophile precursors (Brocksom *et al.*, 2001). It was in this context that the title compound, (I), was investigated (Finelli, 2004). The synthesis of a similar compound, *via* a multi-component reaction, containing a nitrogen atom in place of the oxygen atom has been published (Vieira *et al.* 2007). Herein, the crystal structure determination of (I) is described.

In (I), Fig. 1, both rings are in a distorted half-chair conformation. The ring puckering parameters are: q_2_ = 0.398 (4), 0.372 (4) Å, q_3_ = 0.254 (4), -0.319 (4) Å, QT = 0.472 (4), 0.490 (4) Å, and θ = 236.6 (6), 155.5 (6)° for the *cyclo*-2-ene-1,4-dione and cyclohexene rings, respectively (Cremer & Pople, 1975). With reference to the cyclohexene ring, the *cyclo*-2-ene-1,4-dione and butyldimethylsilyloxy substituents lie to the same side so that the molecule approximates a U-shape; the methyl group lies to the other side of the molecule.

In the crystal molecules are connected through C—H···O interactions that lead to linear supramolecular chains along the *b* axis, Fig. 2. Chains pack with no specific intermolecular interactions between them, Fig. 3.

### Experimental

The catalytic reaction was carried out using 2,6-dimethylcyclohexa-2,5- diene-1,4-dione (83 mg, 0.61 mmol) in anhydrous dichloromethane (1.2 ml) under nitrogen atmosphere at 293 K, and then zinc chloride (91.5 mg, 0.61 mmol) was added. After 40 min stirring, diastereomeric 5-(*tert*-butyl-dimethyl-silyloxy)-pentadiene-2,4 (71 mg, 0.36 mmol) was slowly added. After 20 h stirring the reaction was ended by adding a saturated solution of NaHCO_3_. The organic phase was extracted with dichloromethane, dried with Na_2_SO_4_ and concentrated on a rota-vapor. The product was purified using a silica gel chromatography column with hexane/ethyl acetate (98:2) as the eluent, yielding compound (I) (131 mg, 0.39 mmol). Crystals were grown by slow evaporation from a solution of 15% of acetyl acetate in hexane at 293 K; *M*.pt: 384.3–386.5 K. ^1^H-NMR (CDCl_3_, p.p.m., 400 MHz): δ 6.58 (d, 1H, J = 1.5 Hz); 5.73 (d, 1H, J = 10.1 Hz); 5.63 (ddd, 1H, J = 10.1 Hz, J = 4.4 Hz, J = 3.1 Hz); 3.85 (d, 1H, J = 4.4 Hz); 2.90 (d, 1H, J = 4.9 Hz); 2.43–2.49 (m, 1H); 1.92 (d, 3H, J = 1.5 Hz); 1.45 (d, 3H, J = 7.6 Hz); 1.32 (s, 3H); 0.73 (s, 9H); -0.03 (s, 3H); -0.13 (s, 3H); ^13^C (CDCl_3_, 50 MHz) δ (p.p.m.) 202.1; 197.7; 147.4; 141.1; 133.1; 125.1; 71.6; 54.2; 53.4; 28.8; 25.5; 19.4; 17.8; 17.2; 16.2; -4.6; -5.2. Anal. calcd for C_19_H_30_O_3_Si_1_: C, 68.22; H, 9.04. Found: C, 68.00; H, 9.21.

### Refinement

Carbon-bound H-atoms were placed in calculated positions (C—H = 0.93 to 0.98 Å) and were included in the refinement in the riding model approximation, with *U*_iso_(H) = 1.2–1.5*U*_equiv_(C).

### Crystal data

**Table:**

C_19_H_30_O_3_Si	*F*(000) = 728
*M**_r_* = 334.52	*D*_x_ = 1.127 Mg m^−^^3^
Monoclinic, *P*2/*c*	Melting point: 385.4 K
Hall symbol: -P 2yc	Mo *K*α radiation, λ = 0.71073 Å
*a* = 15.325 (2) Å	Cell parameters from 25 reflections
*b* = 7.1744 (9) Å	θ = 2.8–27.0°
*c* = 17.965 (2) Å	µ = 0.13 mm^−^^1^
β = 93.577 (9)°	*T* = 293 K
*V* = 1971.4 (4) Å^3^	Prism, colourless
*Z* = 4	0.15 × 0.10 × 0.08 mm

### Data collection

**Table:**

Enraf–Nonius CAD-4 MACH 3 diffractometer	*R*_int_ = 0.072
Radiation source: fine-focus sealed tube	θ_max_ = 27.0°, θ_min_ = 2.8°
Graphite monochromator	*h* = −19→19
ω–2θ scans	*k* = 0→9
4451 measured reflections	*l* = 0→22
4305 independent reflections	3 standard reflections every 30 min
1463 reflections with *I* > 2σ(*I*)	intensity decay: 1.4%

### Refinement

**Table:**

Refinement on *F*^2^	Primary atom site location: structure-invariant direct methods
Least-squares matrix: full	Secondary atom site location: difference Fourier map
*R*[*F*^2^ > 2σ(*F*^2^)] = 0.062	Hydrogen site location: inferred from neighbouring sites
*wR*(*F*^2^) = 0.165	H-atom parameters constrained
*S* = 0.93	*w* = 1/[σ^2^(*F*_o_^2^) + (0.0596*P*)^2^] where *P* = (*F*_o_^2^ + 2*F*_c_^2^)/3
4305 reflections	(Δ/σ)_max_ < 0.001
216 parameters	Δρ_max_ = 0.17 e Å^−^^3^
0 restraints	Δρ_min_ = −0.21 e Å^−^^3^

### Special details

**Table:**

Geometry. All s.u.'s (except the s.u. in the dihedral angle between two l.s. planes) are estimated using the full covariance matrix. The cell s.u.'s are taken into account individually in the estimation of s.u.'s in distances, angles and torsion angles; correlations between s.u.'s in cell parameters are only used when they are defined by crystal symmetry. An approximate (isotropic) treatment of cell s.u.'s is used for estimating s.u.'s involving l.s. planes.
Refinement. Refinement of *F*^2^ against ALL reflections. The weighted *R*-factor *wR* and goodness of fit *S* are based on *F*^2^, conventional *R*-factors *R* are based on *F*, with *F* set to zero for negative *F*^2^. The threshold expression of *F*^2^ > 2σ(*F*^2^) is used only for calculating *R*-factors(gt) *etc*. and is not relevant to the choice of reflections for refinement. *R*-factors based on *F*^2^ are statistically about twice as large as those based on *F*, and *R*- factors based on ALL data will be even larger.

### Fractional atomic coordinates and isotropic or equivalent isotropic displacement parameters (Å^2^)

**Table:**

	*x*	*y*	*z*	*U*_iso_*/*U*_eq_	
Si1	0.70796 (7)	0.41802 (16)	0.45217 (6)	0.0549 (4)	
O3	0.76651 (14)	0.3088 (3)	0.51880 (11)	0.0453 (6)	
O1	0.70503 (18)	0.4837 (5)	0.68020 (16)	0.0882 (10)	
O2	0.83093 (19)	−0.1189 (4)	0.57148 (16)	0.0785 (9)	
C5	0.8725 (2)	0.1309 (5)	0.65576 (18)	0.0487 (10)	
H5	0.8723	0.1034	0.7092	0.058*	
C10	0.8338 (2)	0.3308 (5)	0.64516 (18)	0.0455 (9)	
C9	0.8372 (2)	0.3907 (5)	0.56306 (18)	0.0492 (10)	
H9	0.8317	0.5266	0.5602	0.059*	
C8	0.9210 (2)	0.3353 (6)	0.5317 (2)	0.0617 (12)	
H8	0.9339	0.3873	0.4863	0.074*	
C6	0.9670 (2)	0.1243 (6)	0.6364 (2)	0.0596 (11)	
H6	0.9993	0.2009	0.6740	0.072*	
C1	0.7382 (3)	0.3361 (7)	0.6645 (2)	0.0576 (11)	
C7	0.9777 (3)	0.2191 (6)	0.5632 (2)	0.0642 (12)	
H7	1.0279	0.1935	0.5385	0.077*	
C4	0.8100 (3)	−0.0050 (6)	0.6171 (2)	0.0569 (11)	
C2	0.6855 (3)	0.1634 (8)	0.6628 (2)	0.0618 (12)	
C16	0.6517 (2)	0.2288 (6)	0.3974 (2)	0.0602 (11)	
C3	0.7200 (3)	0.0080 (7)	0.6393 (2)	0.0666 (12)	
H3	0.6856	−0.0988	0.6367	0.080*	
C13	0.8858 (2)	0.4707 (6)	0.6949 (2)	0.0721 (13)	
H13A	0.8557	0.5881	0.6940	0.108*	
H13B	0.9429	0.4873	0.6766	0.108*	
H13C	0.8914	0.4244	0.7451	0.108*	
C17	0.5897 (3)	0.3084 (7)	0.3356 (2)	0.1014 (17)	
H17A	0.6225	0.3792	0.3016	0.152*	
H17B	0.5476	0.3880	0.3571	0.152*	
H17C	0.5599	0.2083	0.3092	0.152*	
C12	1.0098 (3)	−0.0693 (6)	0.6413 (2)	0.0899 (15)	
H12A	1.0013	−0.1231	0.6893	0.135*	
H12B	1.0713	−0.0578	0.6348	0.135*	
H12C	0.9835	−0.1481	0.6029	0.135*	
C18	0.5973 (3)	0.1114 (6)	0.4485 (3)	0.1031 (17)	
H18A	0.5694	0.0120	0.4203	0.155*	
H18B	0.5536	0.1884	0.4691	0.155*	
H18C	0.6348	0.0601	0.4882	0.155*	
C14	0.6293 (3)	0.5766 (6)	0.4944 (2)	0.0964 (16)	
H14A	0.5923	0.5059	0.5250	0.145*	
H14B	0.5942	0.6378	0.4556	0.145*	
H14C	0.6608	0.6681	0.5244	0.145*	
C15	0.7796 (3)	0.5561 (7)	0.3940 (2)	0.0972 (17)	
H15A	0.8082	0.6518	0.4239	0.146*	
H15B	0.7451	0.6122	0.3536	0.146*	
H15C	0.8228	0.4759	0.3744	0.146*	
C11	0.5927 (2)	0.1811 (7)	0.6844 (2)	0.1013 (17)	
H11A	0.5651	0.0610	0.6819	0.152*	
H11B	0.5615	0.2653	0.6509	0.152*	
H11C	0.5922	0.2283	0.7344	0.152*	
C19	0.7175 (3)	0.1042 (8)	0.3628 (3)	0.135 (2)	
H19A	0.6879	0.0001	0.3391	0.202*	
H19B	0.7591	0.0599	0.4009	0.202*	
H19C	0.7475	0.1734	0.3264	0.202*	

### Atomic displacement parameters (Å^2^)

**Table:**

	*U*^11^	*U*^22^	*U*^33^	*U*^12^	*U*^13^	*U*^23^
Si1	0.0648 (7)	0.0482 (7)	0.0504 (6)	−0.0054 (6)	−0.0060 (5)	0.0073 (6)
O3	0.0514 (14)	0.0420 (16)	0.0413 (13)	−0.0090 (13)	−0.0070 (11)	0.0005 (12)
O1	0.093 (2)	0.083 (3)	0.090 (2)	0.018 (2)	0.0228 (18)	−0.013 (2)
O2	0.109 (2)	0.051 (2)	0.074 (2)	−0.0013 (18)	0.0003 (17)	−0.0124 (17)
C5	0.057 (2)	0.054 (3)	0.035 (2)	0.001 (2)	0.0004 (18)	0.0003 (19)
C10	0.049 (2)	0.046 (2)	0.041 (2)	−0.001 (2)	−0.0005 (17)	−0.0072 (19)
C9	0.056 (2)	0.044 (2)	0.047 (2)	−0.011 (2)	−0.0034 (19)	0.002 (2)
C8	0.054 (3)	0.081 (3)	0.050 (2)	−0.020 (2)	0.006 (2)	0.012 (2)
C6	0.057 (3)	0.068 (3)	0.053 (2)	0.007 (2)	−0.004 (2)	−0.006 (2)
C1	0.072 (3)	0.061 (3)	0.039 (2)	0.006 (3)	0.000 (2)	−0.004 (2)
C7	0.048 (3)	0.080 (3)	0.064 (3)	−0.007 (2)	0.004 (2)	−0.004 (3)
C4	0.086 (3)	0.040 (3)	0.044 (2)	0.002 (3)	−0.003 (2)	0.006 (2)
C2	0.054 (3)	0.083 (4)	0.049 (2)	−0.005 (3)	0.002 (2)	0.014 (3)
C16	0.062 (3)	0.060 (3)	0.056 (2)	−0.002 (2)	−0.017 (2)	0.001 (2)
C3	0.070 (3)	0.064 (3)	0.066 (3)	−0.020 (3)	0.005 (2)	0.012 (3)
C13	0.086 (3)	0.067 (3)	0.060 (3)	−0.004 (3)	−0.016 (2)	−0.021 (2)
C17	0.110 (4)	0.106 (4)	0.082 (3)	−0.016 (3)	−0.048 (3)	0.015 (3)
C12	0.088 (3)	0.099 (4)	0.082 (3)	0.040 (3)	0.001 (3)	0.008 (3)
C18	0.125 (4)	0.083 (4)	0.097 (4)	−0.044 (3)	−0.032 (3)	0.019 (3)
C14	0.103 (4)	0.077 (4)	0.107 (4)	0.027 (3)	−0.008 (3)	−0.012 (3)
C15	0.104 (3)	0.108 (4)	0.077 (3)	−0.031 (3)	−0.013 (3)	0.050 (3)
C11	0.061 (3)	0.145 (5)	0.100 (4)	−0.007 (3)	0.020 (3)	0.019 (4)
C19	0.129 (5)	0.143 (6)	0.128 (4)	0.038 (4)	−0.026 (4)	−0.080 (4)

### Geometric parameters (Å, º)

**Table:**

Si1—O3	1.649 (2)	C16—C17	1.527 (5)
Si1—C15	1.850 (4)	C16—C18	1.531 (5)
Si1—C14	1.853 (4)	C3—H3	0.9300
Si1—C16	1.858 (4)	C13—H13A	0.9600
O3—C9	1.430 (3)	C13—H13B	0.9600
O1—C1	1.215 (4)	C13—H13C	0.9600
O2—C4	1.215 (4)	C17—H17A	0.9600
C5—C4	1.505 (5)	C17—H17B	0.9600
C5—C6	1.511 (4)	C17—H17C	0.9600
C5—C10	1.559 (5)	C12—H12A	0.9600
C5—H5	0.9800	C12—H12B	0.9600
C10—C1	1.528 (5)	C12—H12C	0.9600
C10—C13	1.533 (4)	C18—H18A	0.9600
C10—C9	1.540 (4)	C18—H18B	0.9600
C9—C8	1.487 (4)	C18—H18C	0.9600
C9—H9	0.9800	C14—H14A	0.9600
C8—C7	1.307 (5)	C14—H14B	0.9600
C8—H8	0.9300	C14—H14C	0.9600
C6—C7	1.500 (5)	C15—H15A	0.9600
C6—C12	1.536 (5)	C15—H15B	0.9600
C6—H6	0.9800	C15—H15C	0.9600
C1—C2	1.478 (6)	C11—H11A	0.9600
C7—H7	0.9300	C11—H11B	0.9600
C4—C3	1.462 (5)	C11—H11C	0.9600
C2—C3	1.315 (5)	C19—H19A	0.9600
C2—C11	1.503 (5)	C19—H19B	0.9600
C16—C19	1.510 (5)	C19—H19C	0.9600
			
O3—Si1—C15	110.43 (15)	C2—C3—C4	123.2 (4)
O3—Si1—C14	109.46 (17)	C2—C3—H3	118.4
C15—Si1—C14	109.1 (2)	C4—C3—H3	118.4
O3—Si1—C16	104.54 (16)	C10—C13—H13A	109.5
C15—Si1—C16	111.4 (2)	C10—C13—H13B	109.5
C14—Si1—C16	111.9 (2)	H13A—C13—H13B	109.5
C9—O3—Si1	124.6 (2)	C10—C13—H13C	109.5
C4—C5—C6	117.7 (3)	H13A—C13—H13C	109.5
C4—C5—C10	108.3 (3)	H13B—C13—H13C	109.5
C6—C5—C10	111.3 (3)	C16—C17—H17A	109.5
C4—C5—H5	106.3	C16—C17—H17B	109.5
C6—C5—H5	106.3	H17A—C17—H17B	109.5
C10—C5—H5	106.3	C16—C17—H17C	109.5
C1—C10—C13	108.8 (3)	H17A—C17—H17C	109.5
C1—C10—C9	107.5 (3)	H17B—C17—H17C	109.5
C13—C10—C9	109.1 (3)	C6—C12—H12A	109.5
C1—C10—C5	111.0 (3)	C6—C12—H12B	109.5
C13—C10—C5	110.6 (3)	H12A—C12—H12B	109.5
C9—C10—C5	109.8 (3)	C6—C12—H12C	109.5
O3—C9—C8	108.8 (3)	H12A—C12—H12C	109.5
O3—C9—C10	110.4 (3)	H12B—C12—H12C	109.5
C8—C9—C10	111.7 (3)	C16—C18—H18A	109.5
O3—C9—H9	108.6	C16—C18—H18B	109.5
C8—C9—H9	108.6	H18A—C18—H18B	109.5
C10—C9—H9	108.6	C16—C18—H18C	109.5
C7—C8—C9	125.0 (4)	H18A—C18—H18C	109.5
C7—C8—H8	117.5	H18B—C18—H18C	109.5
C9—C8—H8	117.5	Si1—C14—H14A	109.5
C7—C6—C5	110.2 (3)	Si1—C14—H14B	109.5
C7—C6—C12	113.0 (3)	H14A—C14—H14B	109.5
C5—C6—C12	115.3 (3)	Si1—C14—H14C	109.5
C7—C6—H6	105.8	H14A—C14—H14C	109.5
C5—C6—H6	105.8	H14B—C14—H14C	109.5
C12—C6—H6	105.8	Si1—C15—H15A	109.5
O1—C1—C2	119.9 (4)	Si1—C15—H15B	109.5
O1—C1—C10	119.7 (4)	H15A—C15—H15B	109.5
C2—C1—C10	120.3 (4)	Si1—C15—H15C	109.5
C8—C7—C6	124.2 (4)	H15A—C15—H15C	109.5
C8—C7—H7	117.9	H15B—C15—H15C	109.5
C6—C7—H7	117.9	C2—C11—H11A	109.5
O2—C4—C3	121.4 (4)	C2—C11—H11B	109.5
O2—C4—C5	124.0 (4)	H11A—C11—H11B	109.5
C3—C4—C5	114.6 (4)	C2—C11—H11C	109.5
C3—C2—C1	119.1 (4)	H11A—C11—H11C	109.5
C3—C2—C11	124.1 (5)	H11B—C11—H11C	109.5
C1—C2—C11	116.7 (5)	C16—C19—H19A	109.5
C19—C16—C17	108.9 (4)	C16—C19—H19B	109.5
C19—C16—C18	108.9 (4)	H19A—C19—H19B	109.5
C17—C16—C18	107.8 (3)	C16—C19—H19C	109.5
C19—C16—Si1	110.5 (3)	H19A—C19—H19C	109.5
C17—C16—Si1	111.0 (3)	H19B—C19—H19C	109.5
C18—C16—Si1	109.6 (3)		
			
C15—Si1—O3—C9	43.4 (3)	C9—C10—C1—C2	97.9 (4)
C14—Si1—O3—C9	−76.6 (3)	C5—C10—C1—C2	−22.1 (4)
C16—Si1—O3—C9	163.3 (2)	C9—C8—C7—C6	−0.4 (6)
C4—C5—C10—C1	48.9 (4)	C5—C6—C7—C8	18.3 (5)
C6—C5—C10—C1	179.8 (3)	C12—C6—C7—C8	148.9 (4)
C4—C5—C10—C13	169.7 (3)	C6—C5—C4—O2	−0.1 (5)
C6—C5—C10—C13	−59.4 (4)	C10—C5—C4—O2	127.1 (4)
C4—C5—C10—C9	−69.8 (4)	C6—C5—C4—C3	179.2 (3)
C6—C5—C10—C9	61.1 (4)	C10—C5—C4—C3	−53.5 (4)
Si1—O3—C9—C8	−98.3 (3)	O1—C1—C2—C3	174.0 (4)
Si1—O3—C9—C10	138.8 (2)	C10—C1—C2—C3	−4.9 (5)
C1—C10—C9—O3	−41.2 (4)	O1—C1—C2—C11	−2.5 (5)
C13—C10—C9—O3	−159.0 (3)	C10—C1—C2—C11	178.6 (3)
C5—C10—C9—O3	79.6 (3)	O3—Si1—C16—C19	−62.5 (3)
C1—C10—C9—C8	−162.3 (3)	C15—Si1—C16—C19	56.7 (4)
C13—C10—C9—C8	79.8 (4)	C14—Si1—C16—C19	179.1 (3)
C5—C10—C9—C8	−41.5 (4)	O3—Si1—C16—C17	176.5 (3)
O3—C9—C8—C7	−109.2 (4)	C15—Si1—C16—C17	−64.3 (3)
C10—C9—C8—C7	12.9 (5)	C14—Si1—C16—C17	58.1 (4)
C4—C5—C6—C7	78.1 (4)	O3—Si1—C16—C18	57.5 (3)
C10—C5—C6—C7	−47.7 (4)	C15—Si1—C16—C18	176.8 (3)
C4—C5—C6—C12	−51.2 (4)	C14—Si1—C16—C18	−60.9 (3)
C10—C5—C6—C12	−177.0 (3)	C1—C2—C3—C4	2.4 (6)
C13—C10—C1—O1	37.1 (5)	C11—C2—C3—C4	178.7 (3)
C9—C10—C1—O1	−81.0 (4)	O2—C4—C3—C2	−151.9 (4)
C5—C10—C1—O1	159.0 (3)	C5—C4—C3—C2	28.7 (5)
C13—C10—C1—C2	−144.0 (3)		

### Hydrogen-bond geometry (Å, º)

**Table:**

*D*—H···*A*	*D*—H	H···*A*	*D*···*A*	*D*—H···*A*
C9—H9···O2^i^	0.98	2.55	3.524 (5)	171

Symmetry code: (i) *x*, *y*+1, *z*.
